# Provider Survey on Burn Care in India

**DOI:** 10.3390/ebj7010003

**Published:** 2025-12-22

**Authors:** Dorothy Bbaale, Priyansh Nathani, Shlok Patel, Anshul Mahajan, Bhavna Chavla, Christoph Mohr, Julia Elrod, Shobha Chamania, Judith Lindert

**Affiliations:** 1 Department of Plastic Surgery, Beit-Cure Children’s Hospital Malawi, Blantyre P.O. Box 31236, Malawi; dorothybbaale@gmail.com; 2 Global Aid in Pediatric Burns Collaboration (GAP-BURNS); priyanshnathani@gmail.com (P.N.); pshlok27@gmail.com (S.P.); anshul.mahajan147@gmail.com (A.M.); mohr.christoph@gmail.com (C.M.);; 3 WHO Collaborating Centre for Research in Surgical Care Delivery in Low- and Middle-Income Countries, Mumbai 400094, India; 4 Byramjee Jeejeebhoy Medical College, Ahmedabad 380016, India; 5 Government Medical College Amritsar, Amritsar 143001, India; 6 International Committee of Red Cross Geneva, 19 Avenue de la Paix, 1202 Geneva, Switzerland; bhavna_lc@yahoo.com; 7 Department of Pediatric Surgery, University Medical Center Mannheim, Heidelberg University, Theodor-Kutzer-Ufer 1-3, 68167 Mannheim, Germany; 8 Choithram Hospital and Research Centre, 14, Manik Bagh Rd., Indore 452014, India; 9 Department of Pediatric Surgery, University Hospital Rostock, Ernst-Heydemann Str. 8, 18057 Rostock, Germany

**Keywords:** burns, India, health system resources, burn care resources

## Abstract

Background: Burns result in approximately 180,000 deaths annually, with the majority occurring in rural regions of Africa and Southeast Asia. This study aimed to assess the available resources, key challenges, and potential solutions in burn care from the perspective of healthcare providers in India. Methods: An online survey was conducted among burn care professionals across India. The survey was disseminated via social media platforms, burn care networks, and hospital representatives. Results: A total of 105 respondents, primarily from tertiary care centers, participated in the survey. Of these, 64.2% were affiliated with government hospitals, and 40.1% served catchment areas extending beyond 300 km. Dedicated burn units were present in 88.0% of government hospitals, compared to 66.9% in non-government facilities. Treatment costs were significantly lower in government hospitals, with 88.8% offering care either free of charge or at minimal cost (*p* ≤ 0.00001). Conclusions: The findings reveal significant gaps in staff training, intensive care monitoring, and infection prevention. Many patients initially seek help from traditional healers, often delaying appropriate treatment and worsening outcomes. Enhancing education, implementing standard monitoring practices, and ensuring adherence to clinical protocols are critical steps toward improving burn care outcomes in India.

## 1. Introduction

Burn injuries have long posed a significant public health challenge, particularly in low- and middle-income countries (LMICs). India is among the most affected, with an estimated 180,000 burn-related deaths and over 1 million burn injuries annually [1].

In 2019, injuries caused by fire, heat, or hot substances resulted in 23,000 deaths and more than 1.5 million disability-adjusted life years (DALYs) in India [2]. Burn injuries in South Asia, including India, often show a seasonal peak in October and November, coinciding with the Diwali festival [3].

Over the years, India has made significant efforts to reduce burn incidence. A key milestone was the launch of the National Programme for Prevention and Management of Burn Injuries 15 years ago. This initiative aimed to increase burn awareness and improve care access—especially in rural areas, where 64% of the population resides and where the burden of burns is disproportionately high [2,3].

Currently, India has 67 registered burn units and provides 1339 dedicated burn beds, fewer than 300 of which are in burn intensive care units (ICUs) [3]. Moreover, only 15% of the population lives within a two-hour drive of a burn center equipped with both an ICU and a skin bank [3].

Despite a gradual increase in burn care facilities, burns remain an under-prioritized public health issue. Many institutions lack the infrastructure, trained personnel, and medical equipment required to deliver quality care [4]. These challenges are further exacerbated by delayed hospital access and financial/resource limitations, contributing to significantly higher mortality rates in LMICs [5]

This study aims to assess the current state of burn care in India from the perspective of healthcare providers. It seeks to identify developments, ongoing challenges, and gaps in human resources, infrastructure, and medical equipment necessary for effective burn care delivery.

## 2. Materials and Methods

Key parameters of human resources, infrastructure, and medical equipment available for burn care were identified according to the PIPES assessment [6]. Items from the Interburns Primary Assessment Tool for Burn Care in Low- and Middle-income countries were incorporated to draft this survey [7]. These two assessments have already been used in a comparable survey on burn care capacity in Africa [5].

This set of core items was used to create a structured, closed-format, self-administered online survey in English, consisting of 45 questions divided into 8 sections. The pilot survey was first reviewed and tested within the GAP-Burns collaborative network and by four independent Indian health professionals.

### 2.1. Patient and Public Involvement

The survey was addressed to healthcare professionals involved in burn care management in Indian healthcare institutions. The survey was posted on the website www.gap-burns.org and distributed through social media, burn-related mailing lists within India, and representatives of hospitals treating burns using the contact lists from the Indian Burns Association and the Association of Plastic Surgeons in India. The survey was available from 1 October 2022 to 31 March 2023.

Facilities were categorized into the following levels of care: primary, secondary, and tertiary. Primary healthcare facilities are usually the first local point of contact for patients and provide basic healthcare services. Secondary healthcare facilities include district hospitals providing a level of care that is intermediate between primary and tertiary healthcare facilities. Tertiary healthcare facilities are university teaching and major referral hospitals that provide specialized and advanced care. The severity of burns was categorized into three groups in this questionnaire: minor (<15% total body surface area), severe (15–60% TBSA), and massive (>60% TBSA).

### 2.2. Ethics and Consent

Ethical approval was granted by the Ethics Committee of Choithram Hospital & Research Centre in Indore, India (CH/EC/Aug/22/23). Informed consent was obtained.

### 2.3. Statistics

Generated data was checked for errors and unified in a database. Statistical analysis was performed using Python (Python 3.11, 2023). Quantitative statistics were expressed as total numbers and percentages, means, and range. Chi-square and *t*-test were applied. A *p*-value ≤ 0.05 was considered significant.

## 3. Results

### 3.1. Origin and Characteristics of the Respondents

**Figure 1:**
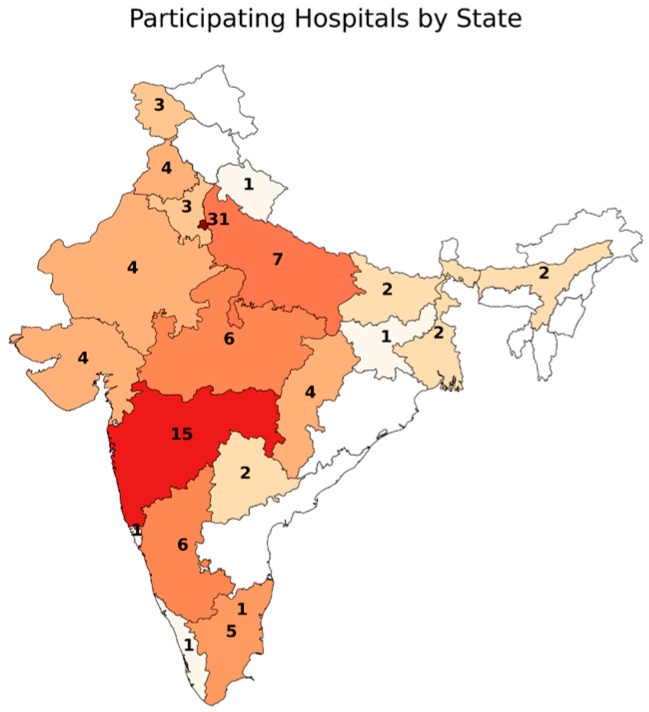
Geographic Distribution of Respondents.

Overall, 105 questionnaires were completed by medical staff working in India. The respondents originated from 24 states with a predominant urban background n = 98 (93.3%); see Table 1 and Figure 1.

Basis information regarding the responding staff can be found in Table 2.

### 3.2. Characteristics of the Healthcare Facility

In total, 94 (89.5%) of all responses originated from tertiary health facilities, 7 (6.7%) from secondary and 4 (3.8%) from primary hospitals. The majority (n = 68, 64.8%) were government-funded, followed by privately funded (n = 34, 32.4%), faith-based (n = 1, 1%), and NGO-funded (n = 2, 1.9%). In total, 53 sites (50.5%) had 4 or more specialists, 49 (46.7) had 1–3, and 3 had none. A total of 49 sites (46.7%) were reportedly teaching hospitals.

#### 3.2.1. Type of Hospital and Geographical Access

In total, 16 (15.2%) hospitals treated patients within a radius of 50 km, 21 (20.0%) within a radius of 51–100 km, 21 (20.0%) within a radius of 101–300 km, and 43 (41.0%) stated that their catchment area was more than 300 km. Most cases were referred from another hospital: 30–70% in 60 institutions (57.1%), more than 70% in 18 places (17.1%), and only 27 (25.7%) saw less than 30% as secondary admissions.

A total of 42 (40.0%) respondents indicated that seasons had an impact on accessibility, while 50 (47.6%) respondents indicated no seasonal impact.

#### 3.2.2. Funding and Payment

Treatment is significantly more often free in government hospitals (*p* ≤ 0.00001). Different payment modalities are demonstrated in Table 3.

#### 3.2.3. Patient Demographics and Pattern of Injuries

A total of 20 institutions (19.0%) see more than 20 new patients per month, and 34 (32.4%) see between 6 and 20 patients. According to the respondents, patients are admitted with the following total body surface area (TBSA) distribution: less than 15% TBSA—37 sites see 1–5 patients, 34 sites see 6–10 patients, and 32 sites see more than 20 patients; 15–60% TBSA—1–5 patients are seen at 42 sites, 6–10 at 28 sites, and more than 20 at 29 sites; and over 60% TBSA—1–5 patients are seen at 54 sites, 6–10 at 22 sites, and more than 20 at 12 sites. Overall, government hospitals are seeing patients with larger total burn areas significantly more often (*p* < 0.0001).

#### 3.2.4. Available Infrastructure

A separate burn unit is available in 85 (80.9%) institutions. Government hospitals have a separate burn ward in 88% of cases (n = 61), which is significantly more often (*p* ≤ 0.0020) than in the other hospitals (66%, N = 24). In 68 (64.1%) places, children are treated in the same ward as adults, and in 35 (33.0%) places, there is a separate pediatric burn unit. A total of 66 (62.8%) institutions have an intensive care unit (ICU) for burn patients, yet 88 (83.8%) can provide intensive care if needed. Similarly, government hospitals have an intensive care unit in 66% of cases (n = 43), whereas the other hospitals have intensive care services available in 55% of cases (N = 20).

**Figure 2:**
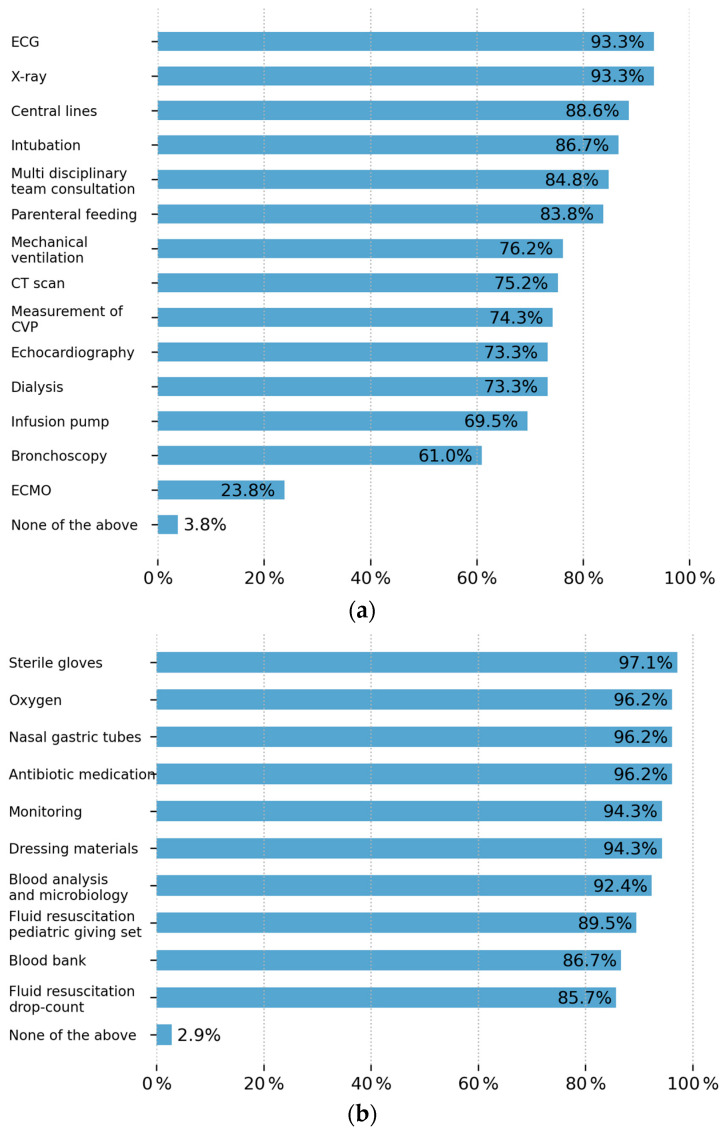
(**a**) Availability of intensive care technology/medical devices. (**b**) Availability of intensive care equipment/consumables.

Figure 2a,b show the constant availability of the following intensive care technology/Medical Devices (a) and equipment/consumables (b).

#### 3.2.5. Surgical Care

An average of four (0–7) operating room days per week are available in the participating centers.

Skin grafting is routinely performed in 104 (98.0%) centers. In total, 57 (54.83%) centers have an electrical dermatome, 26 (24.7%) have a mechanical dermatome, and 84 (80.0%) centers have a Gouillon/Humby knife.

**Figure 3:**
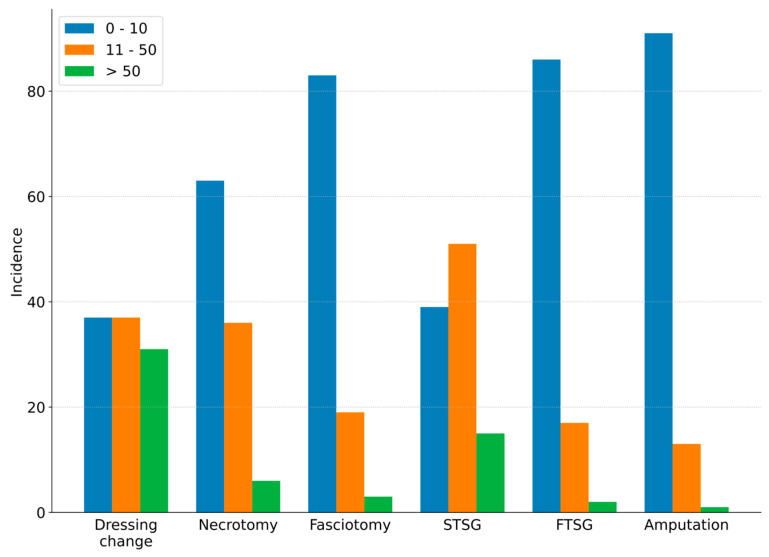
Burn-related surgeries.

Figure 3 shows the estimated number of burn-related surgeries per month.

**Figure 4:**
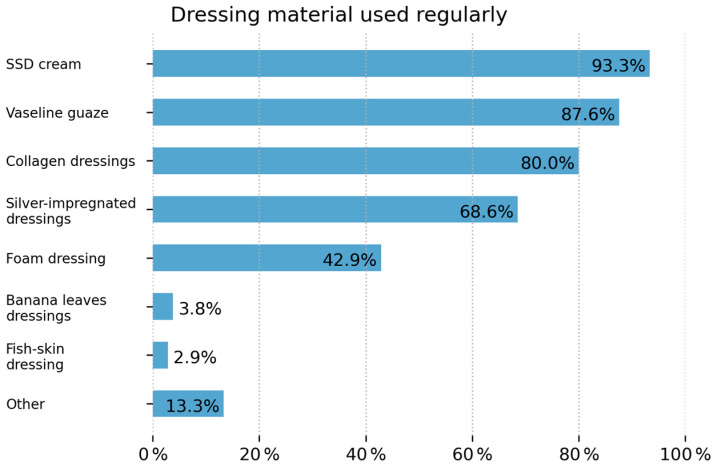
Dressing material used for the care of burn wounds.

Dressing materials used in 98 sites are silver sulfadiazine, 92 petrolatum gauze, 84 collagen dressings, 72 silver-impregnated dressings, 45 foam dressings, 4 banana leaf dressings, 3 fish skin dressings, and 14 use any other dressing material. In total, 69 have access to dermal substitutes with the following distribution of use: 55 Integra^®^ Dermal Regeneration Template, 47 cadaver skin/donor skin, 27 Biodegradable Temporizing Matrix (BTM), and 6 reported the use of xenografts; see Figure 4.

According to respondents, the following circumstances influence poor outcomes; see Table 4.

#### 3.2.6. Potential for Improvement

Respondents agreed (agree and strongly agree, multiple answers possible) that burn care would improve if there was improvement in staff education (n = 67), intensive care monitoring (n = 62), improvement in infection prevention protocols in hospitals (n = 62), free healthcare (n = 58), strengthening of anesthesia (n = 57), increased theater capacity (n = 55), implementation of a blood bank (n = 50), more dressing material (n = 49), and teaching of skin grafting (n = 40).

## 4. Discussion

India, over the last two decades, has taken significant strides to improve burn care management and prevention with the support of the government and different NGOs, particularly via the “National programme for prevention of burn injuries” [8]. However, with its rapidly growing population, it is difficult to ascertain whether these interventions are adequate. Our study attempts to assess this and give an overview of the current situation; however, due to the low response rate, especially from rural areas, it is difficult to have a true depiction of the situation on the ground.

### 4.1. Respondent Characteristics

The survey was predominantly answered by plastic surgeons, with many having over a decade of experience. This preliminary indicates a highly specialized workforce with a high level of experience, which would align with expectations considering India’s global lead in medical education and its historical contributions to reconstructive surgery [9,10]. Although global health has achieved substantial gains over the past 25 years, progress has been uneven. The development of safe, essential, and life-saving surgical and anaesthesia services in low- and middle-income countries (LMICs) is still challenging in many places [11].

Interestingly, approximately half of the institutions had four or more specialists as part of their multidisciplinary team workforce, which significantly differs from the workforce in the African continent, where specialists are few and general practitioners and nursing teams are the backbone of their teams. Nonetheless, the potential for response bias must be acknowledged, as the survey dissemination was significantly skewed towards plastic surgeons practicing in urban centers, especially in tertiary and teaching institutions, and only a minority of respondents were from rural-based institutions [12]. We definitely see there is good availability of intensive care [13,14].

### 4.2. Equipment and Out-of-Pocket Payments

The majority of results captured were mainly from tertiary-level government-funded institutions, which are usually the final destination in the health referral system. It is expected that these institutions should be well equipped in order to provide comprehensive care for patients. Our data suggests that most institutions are well equipped and have constant availability of resources, with only up to 20.0% of consumables regularly missing, which the patients and their families pay for with out-of-pocket payments. It is also worth noting that in 60 of the 68 (88.8%) participating government hospitals, treatment is free or only requires out-of-pocket payment for some basic costs. This is significantly lower in comparison to the African study where the rate of out-of-pocket payments was considerably higher [5], despite the fact that many of our participants were from government facilities. This is most likely due to the lack of universal healthcare coverage in many African countries, whereas in India, public healthcare is free. It is, however, once again difficult to determine if secondary and rural-based facilities are as well equipped due to the low response rate from these facilities.

For institutions that require payments, they are mainly for consumables, surgical interventions, dressing materials, and hospital stay. These costs can potentially leave families with large expenses through out-of-pocket payments.

### 4.3. Infrastructure

Separate burn units are known to improve patient outcomes [7]. Interestingly, more government hospitals reported having separate burn wards and burn intensive care facilities in comparison to private and NGO hospitals. This is most likely due to the associated high running costs and expenses needed in order to operate these facilities. However, over 80% of participating institutions reported that they were able to provide ICU services. These findings significantly differ from our African study, where it was noted that only approximately 35% of our respondents reported having separate burn units, and a mere 21.0% had separate burn ICU facilities. However, despite India having a high ICU bed capacity, units continue to remain severely understaffed, and there is still a significant need for more human resources and equipment [15,16,17].

### 4.4. Advanced Wound Care

India has a rapidly growing wound care market with an annual growth rate of 7.4%—the highest in the world—with many dressing materials being locally manufactured [18]. Many of the respondents indicated that they have access to a wide variety of advanced dressing materials, including dermal skin substitutes. Surprisingly, despite having the first skin bank established in 1978, only 47 locations reported the use of allografts. This is most likely due to the challenges of acceptance amongst the public and medical fraternity, not to mention the high capital costs needed to maintain a skin bank [19,20]. However, this is a significant achievement, as skin banks and the use of skin substitutes are still scarcely available in many African countries [5]. 

### 4.5. Skin Grafting

Skin grafting is routinely performed in 99.0% (N = 104) of the facilities. The remaining facilities that indicated that they do not perform skin grafts are primary facilities, which is in accordance with the NPPMRBI protocol [10]. It was also noted that most facilities were well equipped with Gouillon/Humby knives, with more than half of them having access to electrical dermatomes.

As much as this is significantly higher than our findings in our African study, it is also most likely biased due to our low response rate from rural-based facilities. However, it is important to note as part of the NPPMRBI interventions focused on improving burn care in rural areas, training programs were introduced through the National Academy of Burns India (NABI), for any surgeon wishing to acquire the necessary skills for burn care. Therefore, despite our own response rate from institutions in rural settings, one might be able to assume that skin grafting is also being performed in secondary/district hospital institutions.

### 4.6. Outcomes and Opportunities to Improve

Most respondents cited the main reason for poor outcomes is initial treatment by a traditional healer. These findings also concur with our findings from the African study [5]. Other leading factors cited by respondents for poor outcomes included financial constraints and early discharge, most likely due to the high costs incurred from prolonged hospital stays. The average cost per patient in a tertiary burn unit in India is approximately USD 1060.50, which is a considerable financial burden for the average population [21]. Fear of high costs has been proven to be a factor affecting health-seeking behavior and driving many towards finding solutions in alternative medicine [21,22]. As a solution to this problem, the Indian government is attempting to integrate traditional and alternative medicine providers into the existing government pathway of care [23].

Opportunities for improvement in burn care management were similar to those mentioned in the African study and included increased investment in staff education and training, enhanced intensive care monitoring, and improved infection prevention and control measures in hospitals.

## 5. Conclusions

This survey provides first-hand insights into the availability of human resources, infrastructure, and medical equipment for burn care across the Indian subcontinent. Compared to similar studies conducted in Africa, notable differences have emerged. In India, there is a greater availability of specialized human resources, with burn care teams predominantly led by plastic surgeons. Many institutions also report better access to essential infrastructure and equipment, enabling both surgical interventions and critical care for burn patients. Notably, skin grafting procedures are routinely performed at all participating tertiary and secondary hospitals. There is also easier access to advanced dressing materials and skin substitutes, further supporting improved clinical outcomes. Additionally, out-of-pocket payments are significantly lower in India compared to the African cohort, especially in government hospitals, where many services are subsidized or offered free of charge. However, the survey also highlights a shared challenge between both regions: health-seeking behavior. In both India and parts of Africa, many patients initially seek treatment from traditional healers, contributing to delayed medical care and poorer outcomes.

Areas identified for improvement in India include the following:

- Enhanced staff education and training;
- Improved intensive care monitoring;
- Strengthened adherence to infection prevention protocols.

The progress made in India, particularly through the implementation of the National Programme for Prevention and Management of Burn Injuries (NPPMRBI) and direct government involvement, is highly commendable. These efforts have significantly improved burn care delivery in the country and may serve as a replicable model for other LMICs aiming to strengthen their burn care systems.

## Figures and Tables

**Table 1:** Geographic origin of respondents.

Location	Number				
Delhi	n = 26	Punjab	n = 4	Telangana	n = 2
Maharashtra	n = 15	Rajasthan	n = 4	West Bengal	n = 2
Kamataka	n = 6	Jammu and Kashmir	n = 3	Goa	n = 1
Madhya Pradesh	n = 6	Haryana	n = 3	Jharkhand	n = 1
Uttar Pradesh	n = 6	Tamilnadu	n = 3	Kerala	n = 1
New Delhi	n = 5	Assam	n = 2	Puducherry	n = 1
Chhattisgarh	n = 4	Bihar	n = 2	Uttar Pradesh	n = 1
Gujarat	n = 4	Tamil Nadu	n = 2	Uttarakhand	n = 1

**Table 2:** Characteristics of respondents.

		n	%
Profession n=	Specialist Plastic Surgery	90	85.7
	Specialist General Surgery	6	5.7
	Student	3	2.8
	Physician Assistant	1	1
Years of experience treating burn patients	Less than 2 years	23	21.9
	2–5 years	15	14.3
	6–10 years	10	9.5
	More than 10 years	57	54.3

**Table 3:** Financial cost associated with treatment (multiple choice).

		n	%
Typical payment of treatment for burns (multiple choice)	Patient pays on arrival	18	17.1
	Patients pay later	29	27.6
	Free healthcare for all patients	45	42.9
	Free healthcare for small children	5	4.8
	Free healthcare for special groups (other than children)	12	11.4
	Partly free, some basic costs are covered by the families	27	25.7
	Mainly paid by insurance	7	6.7
If patients need to pay, what they pay for			
	Consumables for dressings	52	49.5
	Consumables for surgical interventions (sutures, blades)	54	51.4
	Fee for intervention (surgery, dressing)	39	37.1
	Fee for hospital stay	49	46.6
	Fee for laboratory	42	40.0
	No extra payment	32	30.5

**Table 4:** Perceived poor outcome stated by respondents (multiple answers possible).

Patient presents late due to previous treatment by traditional healer	n = 81
Patient refuses treatment or leave the hospital early	n = 44
Patient cannot afford treatment (no means for treatment)	n = 41
Overall lack of staff	n = 23
Lack of trained staff	n = 22
Lack of an intensive care unit (defined as constant availability of monitoring, oxygen, fluid management)	n = 15
Lack of dedication of the staff	n = 13
Lack of theater space capacity	n = 12
Lack of dressing material	n = 7
Lack of sedation/anesthesia	n = 5
Lack to perform skin grafting	n = 4
Lack of blood bank	n = 3
Lack of constant power supply	n = 1

## Data Availability

Data can be made available by request to the author team.

## Abbreviations

BTM	Biodegradable Temporizing Matrix
CVP	Central Venous Pressure
DALY	Disability-Adjusted Life Years
HIC	High-Income Country
ICU	Intensive Care Unit
LMIC	Low- and Middle-Income Country
NPPMRBI	National Programme for Prevention, Management and Rehabilitation of Burn Injuries
PIPES	Personnel, Infrastructure, Procedures, Equipment and Supplies-Assessment of Surgery Capacity
STSG	Split Thickness Skin Graft
TBSA	Total Body Surface Area

## References

[1] World Health Organization (WHO) (2023). Fact Sheets. https://www.who.int/news-room/fact-sheets/detail/burns.

[2] Keshri V.R., Abimbola S., Parveen S., Mishra B., Roy M.P., Jain T., Peden M., Jagnoor J. (2023). Navigating health systems for burn care: Patient journeys and delays in Uttar Pradesh, India. Burns.

[3] Tandon R., Agrawal K., Narayan R.P., Tiwari V., Prakash V., Kumar S., Sharma S. (2012). Firecracker injuries during Diwali festival: The epidemiology and impact of legislation in Delhi. Indian J. Plast. Surg..

[4] Sasor S.E., Chung K.C. (2019). Upper Extremity Burns in the Developing World. Hand Clin..

[5] Lindert J., Bbaale D., Mohr C., Chamania S., Bandyopadhyay S., Boettcher J., Katabogama J.B., Alliance B.W., Elrod J. (2023). State of burns management in Africa: Challenges and solutions. Burns.

[6] Markin A., Barbero R., Leow J.J., Groen R.S., Perlman G., Habermann E.B., Apelgren K.N., Kushner A.L., Nwomeh B.C. (2014). Inter-Rater Reliability of the PIPES Tool: Validation of a Surgical Capacity Index for Use in Resource-Limited Settings. World J. Surg..

[7] Potokar T., Bendell R., Phuyal K., Dhital A., Karim E., Falder S., Kynge L., Price P. (2022). The development of the Delivery Assessment Tool (DAT) to facilitate quality improvement in burns services in low-middle income countries. Burns.

[8] Makhija L., Bajaj S., Gupta J. (2010). National programme for prevention of burn injuries. Indian J. Plast. Surg..

[9] Zodpey S., Sharma A., Zahiruddin Q.S., Gaidhane A., Shrikhande S. (2018). Allopathic Doctors in India. J. Health Manag..

[10] Singh V. (2017). Sushruta: The father of surgery. Natl. J. Maxillofac. Surg..

[11] Meara J.G., Leather A.J.M., Hagander L., Alkire B.C., Alonso N., Ameh E.A., Bickler S.W., Conteh L., Dare A.J., Davies J. (2015). Global Surgery 2030: Evidence and solutions for achieving health, welfare, and economic development. Lancet.

[12] Jadhav T., Vissoci J.R.N., Zadey S. (2022). Measuring timely geographical access to surgical care in India: A geospatial modelling study. Lancet Glob. Health.

[13] Ghia C., Rambhad G. (2023). Implementation of equity and access in Indian healthcare: Current scenario and way forward. J. Mark. Access Health Policy.

[14] Kumar V., Venkataraman R., Bajan K., Mehta Y., Govil D., Ramakrishnan N., Zirpe K., Sircar M., Gurav S., Samavedam S. (2022). Intensive Care in India in 2018–2019: The Second Indian Intensive Care Case Mix and Practice Patterns Study. Indian J. Crit. Care Med..

[15] Tirupakuzhi Vijayaraghavan B.K., Nainan Myatra S., Mathew M., Lodh N., Divatia J.V., Hammond N., Jha V., Venkatesh B. (2021). Challenges in the delivery of critical care in India during the COVID-19 pandemic. J. Intensive Care Soc..

[16] Prabu D., Gousalya V., Rajmohan M., Dhamodhar Dinesh M., Bharathwaj V.V., Sindhu R., Sathiyapriya S. (2023). Need Analysis of Indian Critical Health Care Delivery in Government Sectors and Its Impact on the General Public: A Time to Revamp Public Health Care Infrastructure. Indian J. Crit. Care Med..

[17] Kashyap R., Vashistha K., Saini C., Dutt T., Raman D., Bansal V., Singh H., Bhandari G., Ramakrishnan N., Seth H. (2020). Critical care practice in India: Results of the intensive care unit need assessment survey (ININ2018). World J. Crit. Care Med..

[18] Yadav V., Mittal A., Bansal P., Singh S.K. (2019). Regulatory approval process for advanced dressings in India: An overview of rules. J. Wound Care.

[19] Keswani S.M., Mishra M.G., Karnik S., Dutta S., Mishra M., Panda S., Varghese R., Virkar T., Upendran V. (2018). Skin banking at a regional burns centre—The way forward. Burns.

[20] Roberson J.L., Pham J., Shen J., Stewart K., Hoyte-Williams P.E., Mehta K., Rai S., Pedraza J.M., Allorto N., Pham T.N. (2020). Lessons Learned From Implementation and Management of Skin Allograft Banking Programs in Low- and Middle-Income Countries: A Systematic Review. J. Burn. Care Res..

[21] Ahuja R.B., Goswami P. (2013). Cost of providing inpatient burn care in a tertiary, teaching, hospital of North India. Burns.

[22] Alok A. (2020). Problem of Poverty in India. Int. J. Res. Rev..

[23] Josyula K.L., Sheikh K., Nambiar D., Narayan V.V., Sathyanarayana T., Porter J.D. (2016). “Getting the water-carrier to light the lamps”: Discrepant role perceptions of traditional, complementary, and alternative medical practitioners in government health facilities in India. Soc. Sci. Med..

